# Supplementary material for: AAV Capsid Screening for Translational Pig Research Using a Mouse Xenograft Liver Model
Source: bioRxiv. 2024 May 29:2024.05.29.596409. Preprint. [Version 1] doi: 10.1101/2024.05.29.596409 (PMC11160762; doi:10.1101/2024.05.29.596409)
Supplement: Supplement 1 [file media-1.pdf]

donor (gender)	FRGN mice transplanted	porcine FIX positive	% successful repopulation
Sus domesticus, fresh (female)	**5 /** 4	**2** / 0	**40%** / 0%
Gottingen Minipig, cp (male)	**3 /** 14	**1**/ 2	**33%**/ 14%
Sus domesticus, cp (female)	**14 /** -	**4** / -	**29%** / -
total	**22** / 18	**7** / 2	**31%** / 11%

Table S1: *Summary of xenotransplantation and repopulation success in FRGN mice using porcine hepatocytes (fresh or cp = cryopreserved). Hepatocytes form Gottingen Minipig were aquired commercially where hepatocytes form Sus domesticus were isolated in-house. Treatment of number of recipients:* ***bold*** *= no Ad:uPA; normal = Ad:uPA.*

mouse ID	pre-depletion (%)	post-depletion (%)	body weight (g)	liver weight (g)
noID	92.4	97.2	24.9	3.6
2TR	97.1	99.6	26.2	-
2BR	95.6	99.6	28	5.1

Table S2: *FACS quantification of porcine hepatocytes after hepatocyte extraction before and after depletion of mouse cells using MACS.*

serotype	origin
AAV1	human/non-human primate
AAV2	human
AAV3	human
AAV4	non-human primate
AAV5	human
AAV6	AAV1 and AAV2 hybrid
AAV7	rhesus macaque
AAV8	rhesus macaque
AAV9	non-human primate
AAV10	cynomolgus monkey
AAV11	cynomolgus monkey
AAV1_9mt30	engineered
AAV1_9mt76	engineered
AAV1_9mt100	engineered
AAV2retro	engineered
AAV9AA22	engineered
AAV9AA272	engineered
AAV9W22A	engineered
rh8	rhesus macaque
rh10	rhesus macaque
rh20	rhesus macaque
rh43	rhesus macaque
sh10	engineered
Anc80	engineered
bb2	babboon
DJ	shuffled
HN1	engineered
HN2	engineered
HN3	engineered
hu11	human
hu13	human
hu37	human
KP1	in vivo directed evolution
KP2	in vivo directed evolution
KP3	in vivo directed evolution
LK03	in vivo directed evolution
NP40	shuffled
NP59	shuffled
PHPB	engineered
PHPeB	engineered
PHPS	engineered
Pig	NA
R585E	engineered
R585E9_2	engineered
2G9	engineered
2i8	domain swapping
7m8	engineered

Table S3: *47 serotypes of the AAV-serotype library and their corresponding origin, natural or engineered*

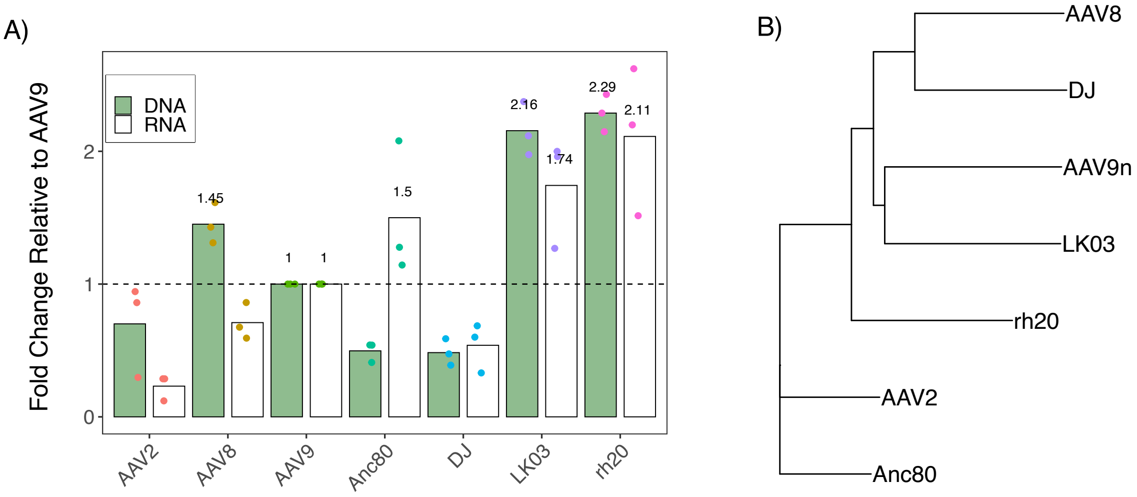

Figure S1: *Selected AAV capsids for in vitro testing. A) Capsids from AAV2, AAV8, AAV9, AAVAnc80, AAVDJ, AAVLK03, and AAVrh20 were chosen based on their efficiency or as controls for further in vitro comparison using primary porcine hepatocytes. B) Phylogenetic tree representing the genetic relation of the seven chosen capsids.*

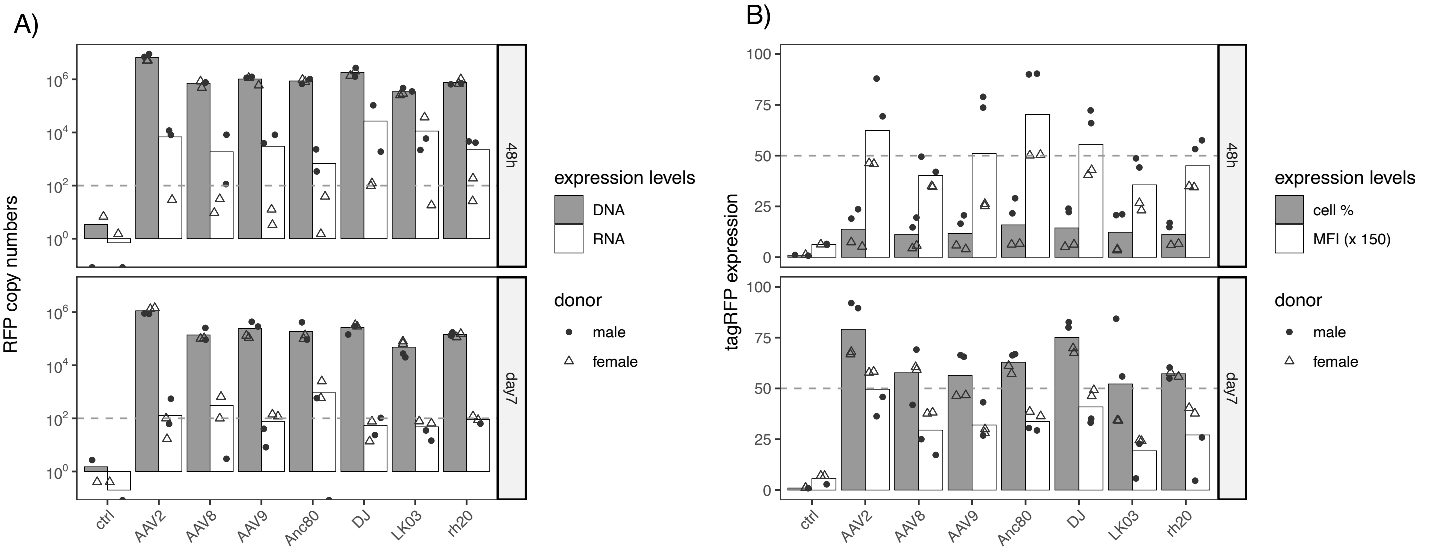

Figure S2: *absolute transduction and expression efficiency of seven selected rAAV serotypes in primary porcine hepatocytes (donors = 2) transduced post-isolation (MOI 20,000 vg/cell) and cultured in vitro for 48 hours or 7 days. A) Relative mean DNA and RNA amounts post-transduction in primary porcine hepatocytes measured using qPCR and RT-qPCR, respectively. B) Relative mean of tagRFP-positive single cells and Median Fluorescent Intensity (MFI) post-transduction in primary porcine hepatocytes measured using fluorescence activated cell sorting (FACS). Dashed line represents 50% tagRFP positive cells, 7500 MFI, and* 10^2 *tagRFP copy numbers per 100 ng DNA or 1200 ng RNA.*

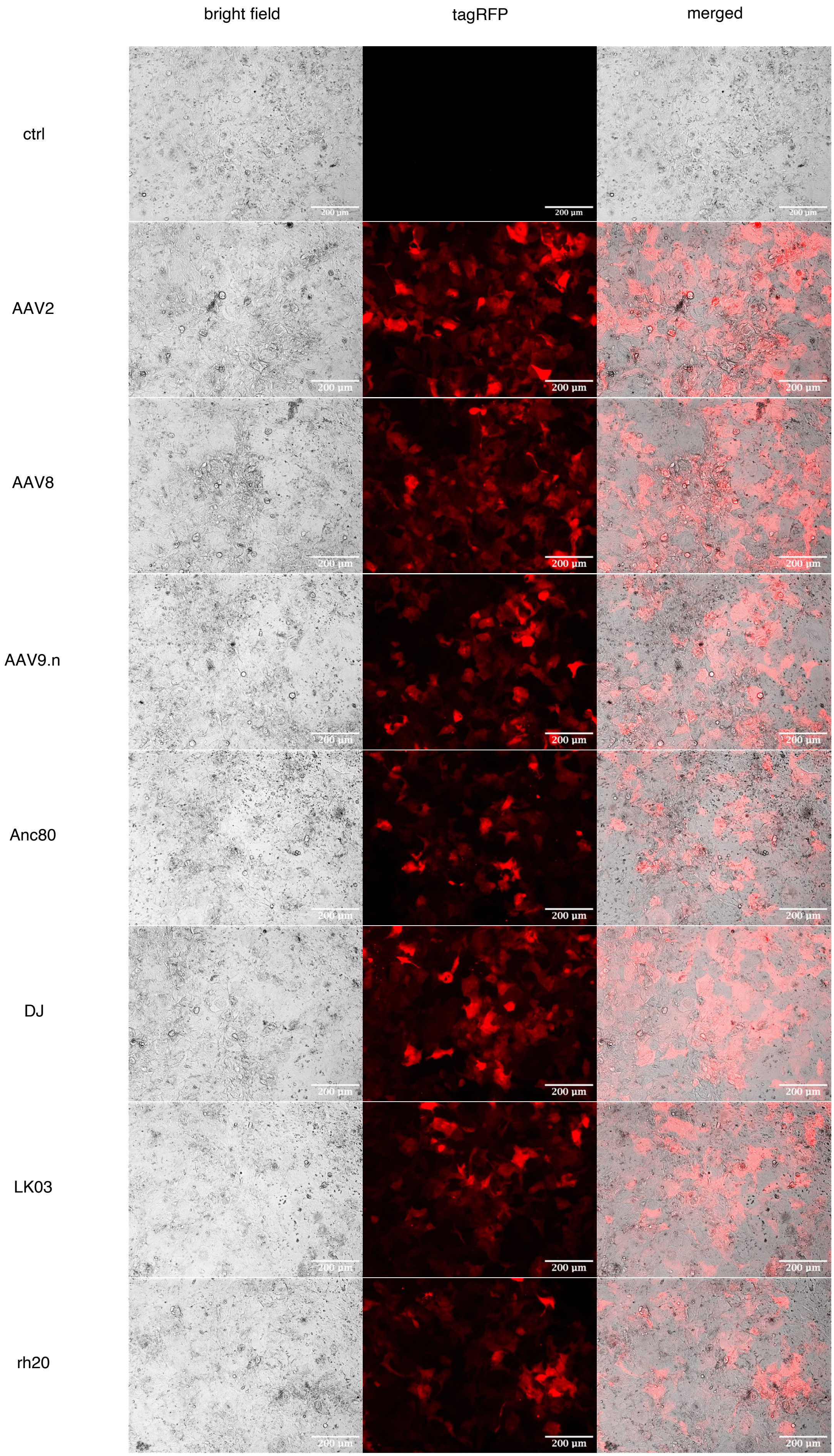

Figure S3: *Visualization of tagRFP expression in primary porcine hepatocytes transduced with selected AAV-serotypes, seven days post-transduction. Scale bars, 200 µm.*
